# Modulatory Effects of the Er,Cr:YSGG—Diode Laser Combination Irradiation on Human Osteoblasts—Might Be Relevant for Periapical Bone Regeneration?

**DOI:** 10.3390/ijms27219839

**Published:** 2026-11-03

**Authors:** Alexandra Ripszky, Isaac Kably, Cristina Neculau, Bianca Voicu Balasea, Izet Melis, Sebastian Bancu, Ana Cernega, Radu Radulescu, Silviu Pituru

**Affiliations:** 1 The Interdisciplinary Center for Dental Research and Development, “Carol Davila” University of Medicine and Pharmacy, 19-21 Jean Louis Calderon, 030167 Bucharest, Romania; alexandra.ripszky@umfcd.ro (A.R.); izet.melis@umfcd.ro (I.M.); silviu.pituru@umfcd.ro (S.P.); 2 Department of Biochemistry, Faculty of Dental Medicine, “Carol Davila” University of Medicine and Pharmacy, 17-23 Plevnei Street, 020021 Bucharest, Romania; sebastian.bancu@drd.umfcd.ro (S.B.); radu.radulescu@umfcd.ro (R.R.); 3 Asociacion Mexicana de Laser y Medicina Integrative, Ciudad de México 07850, Mexico; dr@kably.mx; 4 Department of Organization, Professional Legislation and Management of the Dental Office, Faculty of Dental Medicine, “Carol Davila” University of Medicine and Pharmacy, 17-23 Plevnei Street, 020021 Bucharest, Romania; ana.cernega@umfcd.ro

**Keywords:** photobiomodulation, Er,Cr:YSGG laser, diode laser, osteoblasts, alkaline phosphatase, periapical bone regeneration

## Abstract

This in vitro study evaluated how Er,Cr:YSGG irradiation at three energy levels (1, 3, and 6 J/cm^2^), 810 nm diode irradiation, and their combinations can influence human osteoblast viability and osteogenic activity. Human osteoblasts (hOB) were assigned to eight groups: control, diode, Er,Cr:YSGG at 1, 3, and 6 J/cm^2^, and each Er,Cr:YSGG dose followed immediately by diode irradiation. 24 h after irradiation, metabolic activity (MTT), membrane integrity (LDH), nitric oxide (NO) production, and alkaline phosphatase (ALP) activity were assessed, and cell morphology was tracked by holographic live-cell imaging. Cell viability and membrane integrity were preserved across all protocols (MTT *p* = 0.325; LDH *p* = 0.057; both n.s.), and live-cell imaging showed osteoblast morphology and proliferation. Diode irradiation significantly increased ALP activity versus control (12.96 vs. 5.40 IU/L; *p* = 0.011), confirming a biostimulatory osteogenic effect, whereas Er,Cr:YSGG at 6 J/cm^2^ almost completely suppressed ALP activity (0.42 IU/L; *p* < 0.001 vs. diode). Both Er,Cr:YSGG 3 J + diode and 6 J + diode combinations significantly attenuated the diode-induced ALP increase and NO elevation in a dose-dependent manner. No protocol was cytotoxic. The 810 nm diode laser was the most osteogenically favorable condition, whereas higher-energy Er,Cr:YSGG irradiation suppressed ALP activity and, when combined with the diode, attenuated its osteogenic effect.

## 1. Introduction

Periapical bone regeneration after endodontic treatment is coordinated by osteoblasts—specialised mesenchymal cells responsible for producing, depositing, and mineralising the organic bone matrix. A key player in this mineralisation is tissue-nonspecific alkaline phosphatase (ALP), a membrane-bound enzyme that catalyses the breakdown of inorganic pyrophosphate (PPi), an inhibitor of mineralisation. This process releases inorganic phosphate (Pi), which is crucial for the hydroxyapatite crystal assembly [1,2,3].

ALP functions through two main mechanisms: it removes PPi, which otherwise binds calcium and blocks abnormal calcification, and it supplies Pi needed for calcium phosphate formation within matrix vesicles that originate from osteoblast membranes [4,5,6]. In addition to its enzymatic activity, ALP plays a major role in regulating osteogenic differentiation by influencing the expression of runt-related transcription factor 2 (RUNX2). RUNX2 is the key transcription factor that directs osteoblast lineage commitment and promotes the production of bone matrix proteins like type I collagen, osteocalcin, and osteopontin [4,5,6]. This regulatory role makes ALP more than just a biochemical marker because it acts as a functional regulator of osteoblastic differentiation and thus significantly impacts bone healing potential [4,5,6].

The Erbium, Chromium: Yttrium–Scandium–Gallium–Garnet (Er,Cr:YSGG) laser, operating at 2780 nm, it is seen more and more often as a promising adjunct in endodontic disinfection protocols due to its high absorption coefficients in water and hydroxyapatite—the principal constituents of the dental hard tissues [7,8]. Through hydrokinetic activation, the laser generates localized micro-explosions as water molecules within the dentinal matrix absorb photon energy, producing thermomechanical ablation that can penetrate dentinal tubules to depths exceeding 500 μm—substantially surpassing the 130 μm penetration limit of sodium hypochlorite, the conventional chemical disinfectant [7,8]. Recent randomised clinical trials have demonstrated that dual-wavelength protocols that combine Er,Cr:YSGG (2780 nm) with diode lasers achieve bacterial reduction rates approaching 99% in infected root canal systems, with superior clinical outcomes in teeth with apical periodontitis [7,8]. However, despite compelling evidence of antimicrobial efficacy, a fundamental biological question remains inadequately addressed: how does laser energy transmitted through the apical foramen, lateral canals, or via thermal conduction affect the periapical osteoblast population responsible for post-treatment bone regeneration? The answer to this question has profound implications for clinical parameter selection, as optimal endodontic laser protocols must balance microbial elimination with the preservation—or, ideally, the enhancement—of osteogenic differentiation in the periapical tissues.

**Figure 1:**
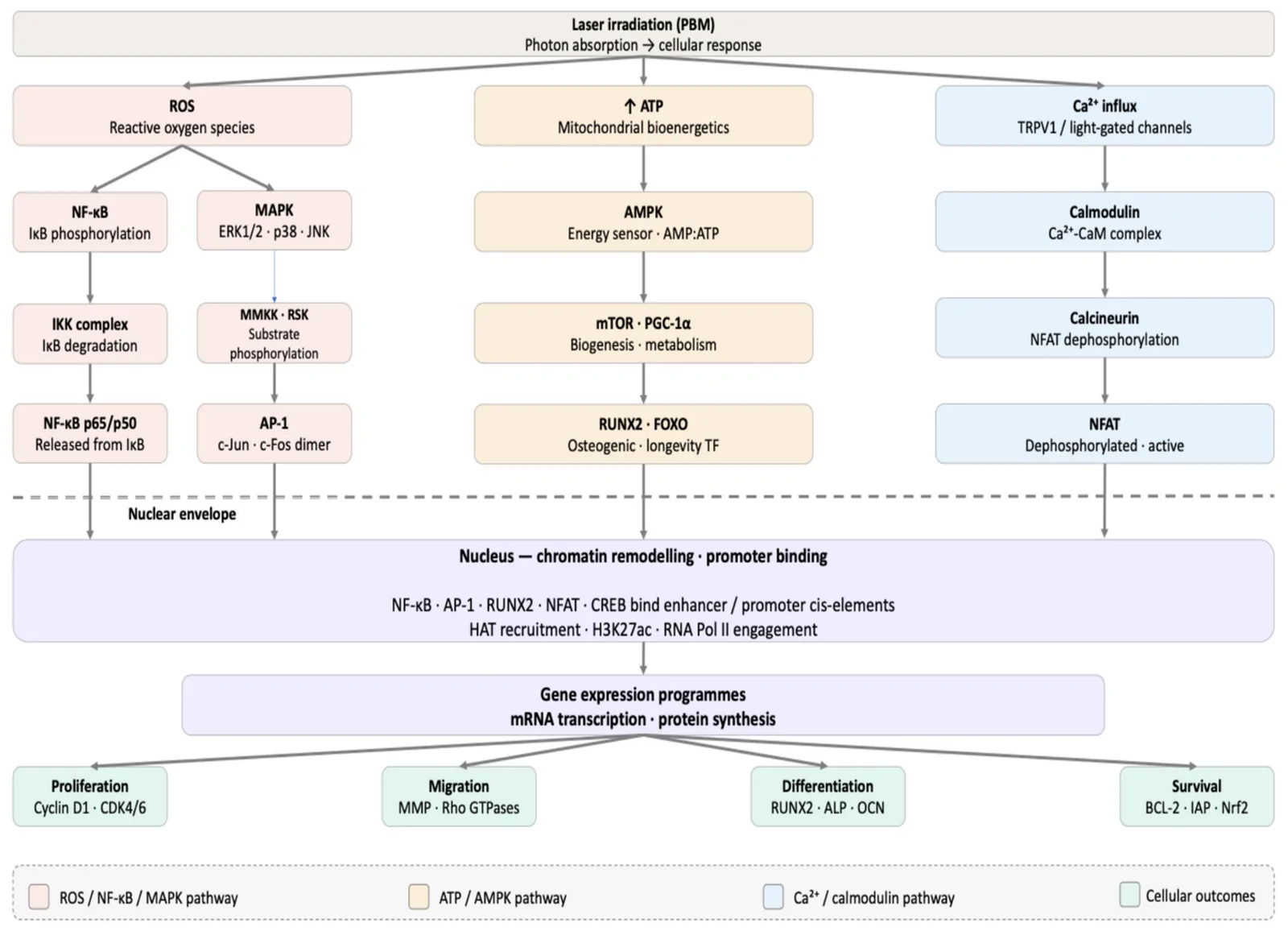
**Activated Intracellular Signaling Cascades by Photobiomodulation (PBM) in Osteoblasts: From Photon Absorption to Gene Expression and Cellular Outcomes.** Sub-ablative laser irradiation activates three interconnected signaling axes that converge on nuclear transcription factors. **Axis I (ROS/NF-κB/MAPK):** photon absorption by cytochrome c oxidase generates a transient, controlled ROS burst. ROS act as second messengers that (1) activate the IKK complex, leading to IκB phosphorylation and degradation and the release of NF-κB p65/p50, and (2) activate MAPK cascades (ERK1/2, p38, JNK), which, via MKK and RSK, activate AP-1 (c-Jun/c-Fos). **Axis II (ATP/AMPK):** enhanced oxidative phosphorylation raises ATP and lowers the AMP:ATP ratio, modulating AMPK. Through mTOR and PGC-1α, this links bioenergetics to mitochondrial biogenesis, metabolic adaptation and the activation of RUNX2 and FOXO. **Axis III (Ca^2+^/calmodulin):** activation of light-gated channels (e.g., TRPV1) triggers Ca^2+^ influx and the formation of the Ca^2+^-CaM complex. This activates calcineurin, which dephosphorylates NFAT, and CaM-dependent kinases, which activate CREB. In the nucleus, NF-κB, AP-1, RUNX2, NFAT and CREB bind enhancer and promoter cis-elements, recruit HATs (H3K27ac) and engage RNA Pol II. The resulting gene expression programs drive four outcomes: proliferation (Cyclin D1, CDK4/6), migration (MMPs, Rho GTPases), differentiation (RUNX2, ALP, OCN) and survival (BCL-2, IAP, Nrf2) [9,12,13,14,15,16,17,18].

Interactions between light and tissue at sub-ablative energy levels initiate photobiomodulation (PBM), a series of photochemical reactions triggered when photons are absorbed by endogenous chromophores, mainly cytochrome c oxidase (CO), the last enzyme in the mitochondrial electron transport chain (complex IV) [9]. This enzyme has heme iron and copper redox centers that absorb photons in the red and near-infrared spectra (600–1000 nm) [9]. When photons are absorbed, they displace the inhibitory nitric oxide (NO) from cytochrome c oxidase’s catalytic site, restoring electron flow and relieving competitive inhibition of oxygen reduction [9]. This event triggers cellular responses: increased mitochondrial membrane potential (ΔΨm), higher ATP production through oxidative phosphorylation, brief generation of reactive oxygen species (ROS), and the release of intracellular calcium by modulating light-sensitive ion channels (like TRPV1) [9]. These energy-related effects activate key signalling pathways, as follows: ROS stimulate NF-κB and MAPK pathways; ATP boosts AMPK; calcium influx activates calmodulin-dependent cascades. These molecular events will lead to the mobilisation of transcription factors into the nucleus, where they trigger specific gene programs involved in cell growth, migration, differentiation, and survival (Figure 1) [9].

One of the key features of photobiomodulation—and central to this study—is represented by its biphasic dose–response [9]. This nonlinear pattern, often represented by the Arndt–Schulz curve, indicates that cellular responses do not follow the Bunsen–Roscoe law of reciprocity [10,11]. Instead, there is a therapeutic window where low to moderate light doses (typically 1–20 J/cm^2^ for visible and near-infrared light) stimulate cellular activity, while too low doses have no effect and too high doses can inhibit specific molecular processes or cause cytotoxicity [10,11].

The molecular bases of this biphasic behavior are directly related to the dual nature of ROS. At physiological concentrations (nM to low μM range), ROS act as essential second messengers that activate pro-survival and pro-differentiation signaling cascades. However, excessive ROS alter the redox balance, overwhelming the endogenous antioxidant defence systems (superoxide dismutase, catalase, glutathione peroxidase), triggering oxidative damage to key proteins, membrane lipids and nucleic acids, mitochondrial permeability transition pore (mPTP) opening, cytochrome c release, and activation of pro-apoptotic caspase cascades [9,12,13,14,15]. This dose-dependent switch in ROS function, from signaling molecule to cytotoxic agent, provides an open window for understanding why identical total energy densities delivered at different irradiances or pulse structures can produce divergent biological outcomes [9,12,13,14,15].

This study aimed to assess in vitro how Er,Cr: YSGG laser irradiation at three different energy levels, diode laser irradiation, and their combinations may influence the viability and function of human osteoblasts. Specifically, we examined ALP activity as an indicator of osteogenic differentiation to evaluate the safety and osteogenic potential of these laser protocols in a clinically relevant model for periapical bone regeneration following endodontic treatment.

## 2. Results

### 2.1. Holomonitor Live-Cell Imaging

**Figure 2:**
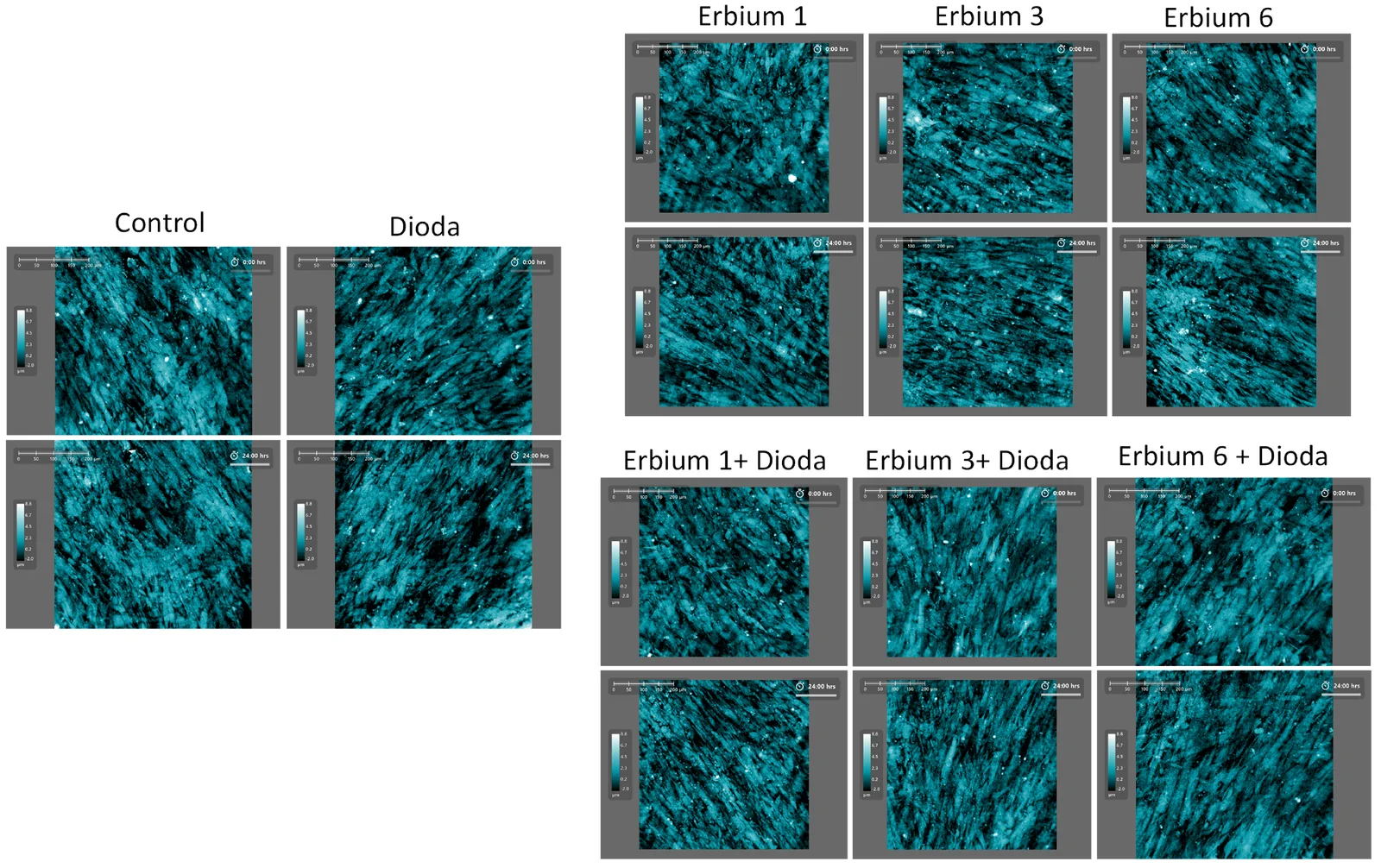
Representative HoloMonitor M4 images of human osteoblasts (hOB) at 0 h and 24 h after irradiation with the Er,Cr:YSGG laser (2780 nm, 600 µm MZ6 tip, 0.25–1.5 W, 15 Hz) alone or combined with diode laser, across all experimental groups (control, diode, Er,Cr:YSGG 1/3/6 J, Er,Cr:YSGG 1/3/6 J + diode).

HoloMonitor live-cell imaging (Figure 2) revealed that osteoblasts maintained normal morphology, adhesion, and proliferative behavior over the 24 h observation period following laser irradiation. Compared with the control group, no evident reduction in cell density or morphological signs of cellular damage were observed in any of the laser-treated groups, indicating preserved cellular integrity and supporting the absence of cytotoxic effects detected in the biochemical assays.

### 2.2. Cellular Metabolic Activity/Viability (MTT)

**Figure 3:**
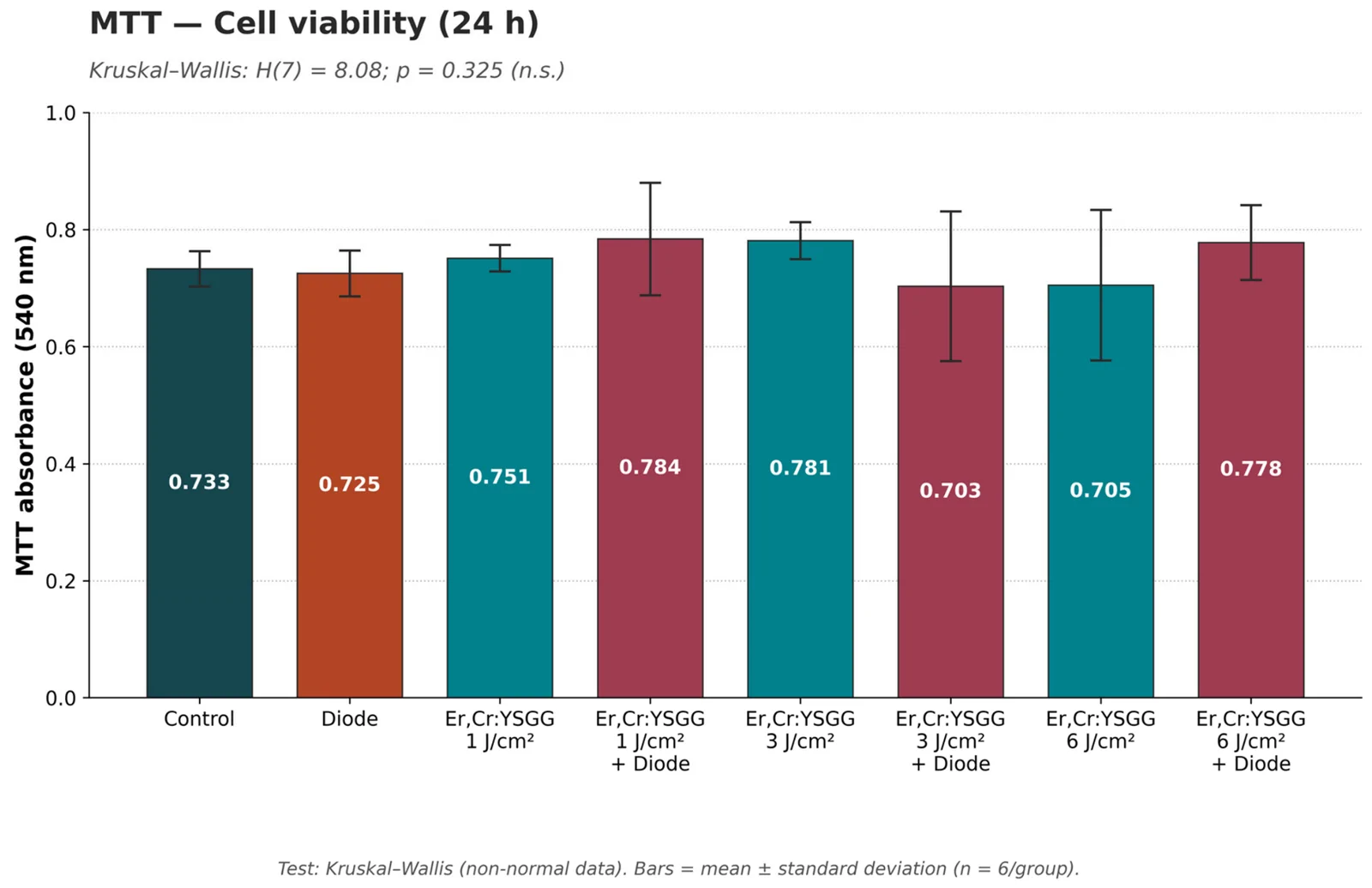
Cell viability by MTT assay (mean ± SD). No significant differences (n.s.).

The Kruskal–Wallis test showed no significant differences in MTT absorbance across groups (H(7) = 8.08, *p* = 0.325). All conditions maintained viability within ~10% of the Control value (0.733 OD), confirming that the differences observed in ALP cannot be attributed to loss of viable cells (Figure 3).

### 2.3. Lactate Dehydrogenase (LDH)—Cytotoxicity

**Figure 4:**
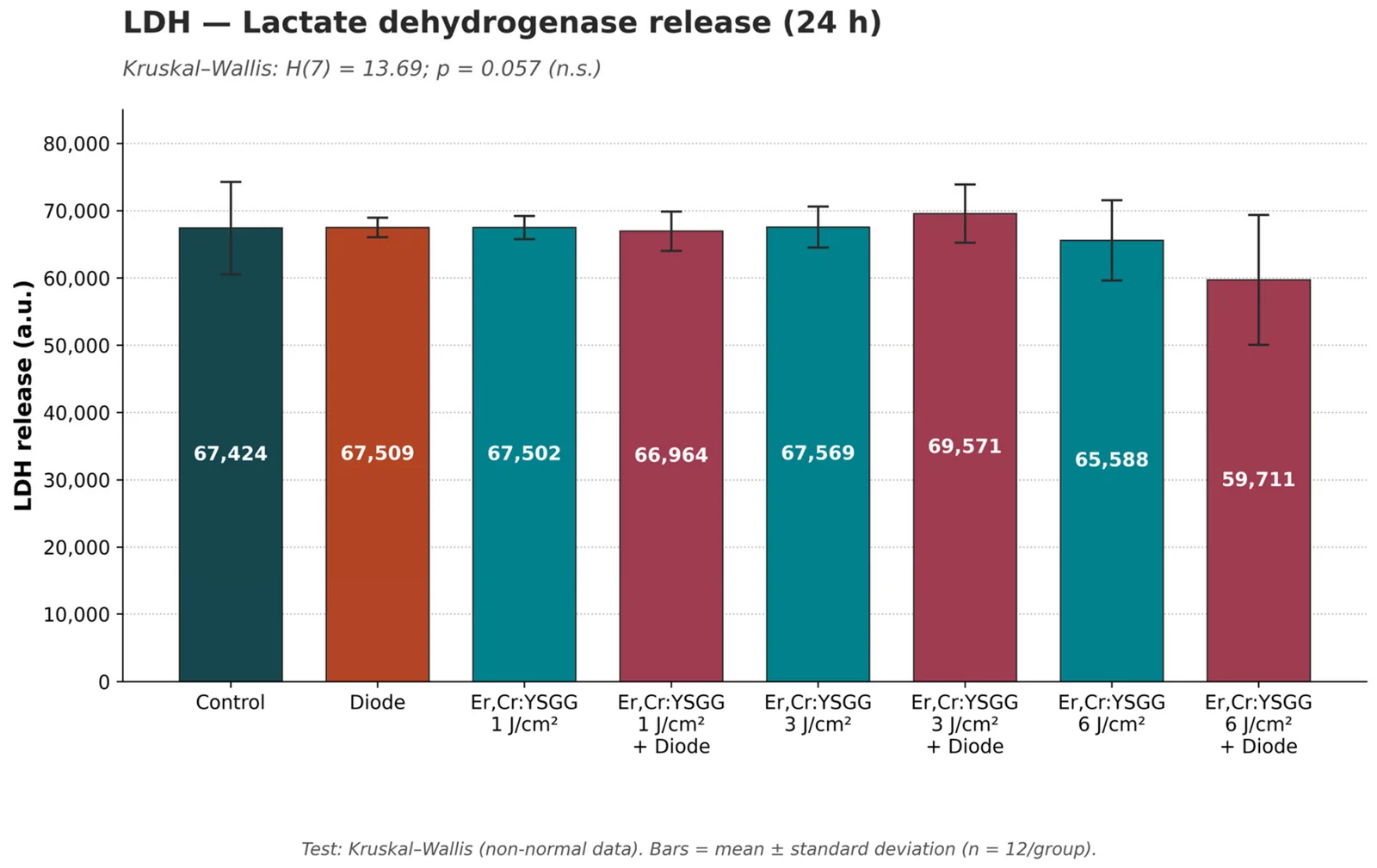
LDH levels across experimental groups (mean ± SD). Differences do not reach statistical significance (n.s.).

The Kruskal–Wallis test did not reveal statistically significant differences between groups (H(7) = 13.69, *p* = 0.057), indicating that none of the irradiation conditions produced detectable membrane damage. Group means ranged from 59,711 to 69,571 AU, with the lowest value observed in the Er,Cr:YSGG 6 J + Diode group, (Figure 4).

### 2.4. Nitric Oxide (NO)

**Figure 5:**
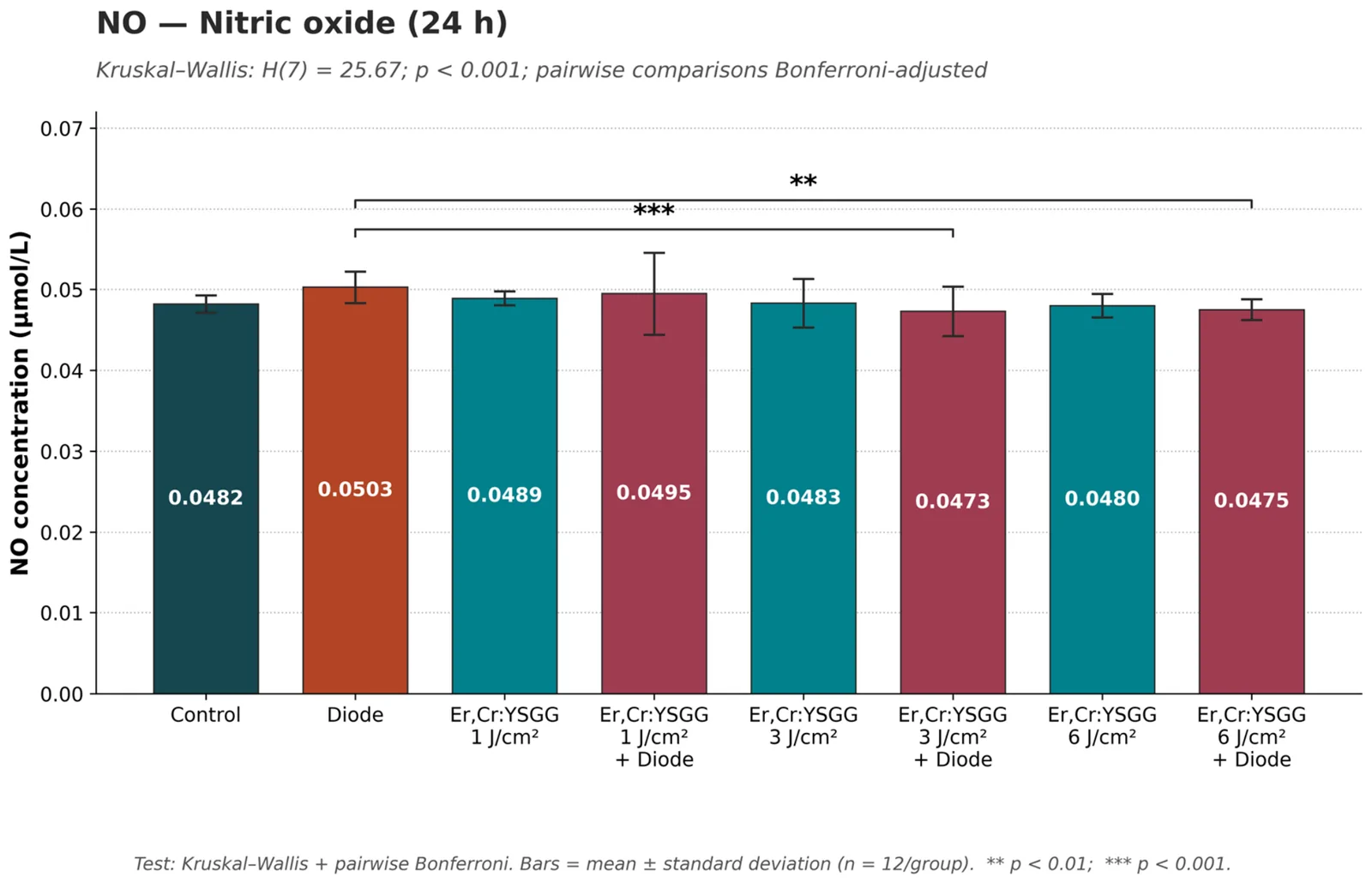
NO concentration across experimental groups (mean ± SD). Stars indicate significance vs. Diode. (Kruskal–Wallis + Bonferroni).

The Kruskal–Wallis test revealed a strong global effect (H(7) = 25.67, *p* < 0.001). NO levels were highest in the Diode group (0.05033 µmol/L) and were significantly reduced by combination with Er,Cr:YSGG at 3 J and 6 J (Er,Cr:YSGG 3 J + Diode: 0.04733 µmol/L, adjusted *p* = 0.0009; Er,Cr:YSGG 6 J + Diode: 0.04750 µmol/L, adjusted *p* = 0.0017) (Figure 5). The remaining pairwise comparisons did not reach statistical significance after Bonferroni correction. The convergence between the ALP and NO findings—both showing attenuation of the Diode effect by Er,Cr:YSGG pre-treatment at medium and high energies—suggests a shared mechanism of cellular response modulation (Figure 5).

### 2.5. Alkaline Phosphatase (ALP)

**Figure 6:**
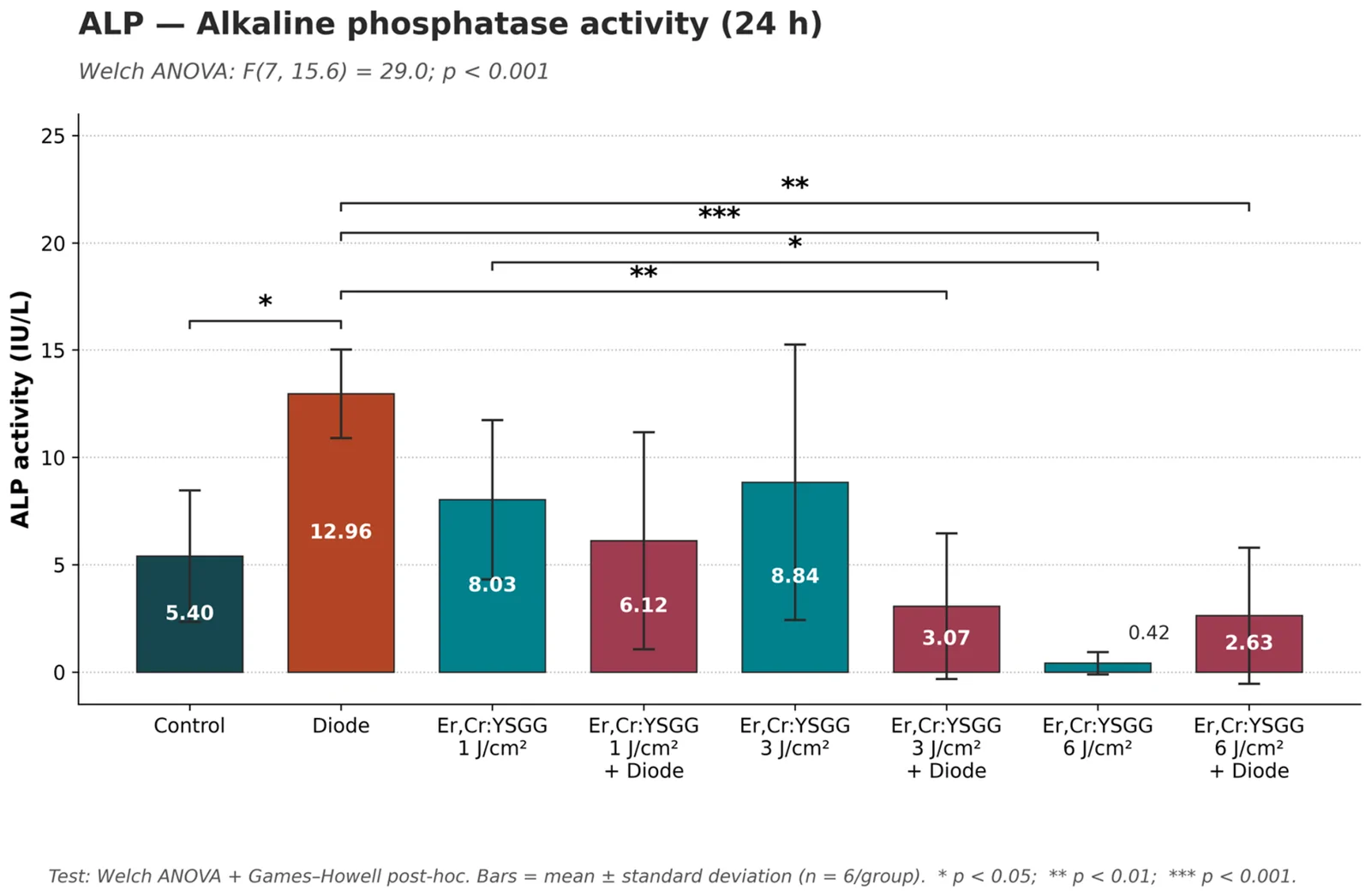
ALP activity across experimental groups (mean ± SD). Stars indicate significance vs. Diode (Games–Howell).

Welch’s ANOVA revealed a strong effect of the experimental conditions on ALP activity (F(7, 15.6) = 29.0, *p* < 0.001) (Figure 6). The Diode group showed significantly higher ALP than Control (12.96 ± 2.06 vs. 5.40 ± 3.06; *p* = 0.011), confirming the biostimulatory effect of the diode laser on osteoblastic differentiation (Figure 6). Conversely, Er,Cr:YSGG irradiation at 6 J strongly suppressed ALP compared with Diode (0.42 ± 0.52; *p* < 0.001), while the Er,Cr:YSGG 3 J + Diode and Er,Cr:YSGG 6 J + Diode combinations significantly attenuated the Diode-induced stimulation (3.07 ± 3.39, *p* = 0.004; and 2.63 ± 3.17, *p* = 0.002, respectively) (Figure 6).

## 3. Discussion

The present in vitro study investigated the effects of Er,Cr:YSGG laser radiation at three energy levels (Erbium 1, 3 and 6), diode laser irradiation, and their combinations on primary osteoblast cultures, evaluating cell metabolic activity/viability (MTT), citotoxicity (LDH), nitric oxide (NO) production, and alkaline phosphatase (ALP) activity as a functional marker of osteogenic differentiation. The main finding was that none of the laser protocols—applied individually or in combination—induced statistically significant changes in any of the four outcome parameters compared with the untreated control, after Bonferroni correction. However, significant differences were observed between the groups, particularly in the NO and ALP parameters, and these contrasts reveal biologically significant interactions between wavelength, energy level, and combined exposure that require careful interpretation.

Neither the MTT assay (Figure 3) nor the LDH release assay (Figure 4) reached statistical significance, indicating that osteoblast metabolic activity and plasma membrane integrity were preserved across all experimental conditions. From a clinical safety perspective, this finding is reassuring, even at the highest energy level of the tested erbium (Er,Cr:YSGG 6) and in the combined condition Er,Cr:YSGG 6 + diode, which produced the largest numerical reduction in LDH values compared with the control. However, no statistically significant cytotoxic threshold was exceeded. Maintenance of cell viability across all protocols is consistent with the principle that sub-ablative laser energy densities at the periapical level, attenuated by dentine and cement, are unlikely to induce the cytotoxic effects for resident osteoblast populations. These results align with previous studies showing that the Er,Cr:YSGG irradiation parameters used in contemporary endodontic protocols do not compromise the viability of periapical cells when applied at recommended energy settings [19,20,21].

Also, no treatment group differed significantly from the control regarding the NO release (Figure 5). The post hoc analysis revealed two specific contrasts between treatments: Diode vs. Er,Cr:YSGG 3 + Diode (*p* = 0.0013, **) and Diode vs. Er,Cr:YSGG 6 + Diode (*p* = 0.0026, **) (Figure 5). In both cases, the results indicated that Er,Cr:YSGG 3 and Er,Cr:YSGG 6 irradiation, combined with diode laser exposure, attenuated the NO elevation induced by diode irradiation alone. Although the absolute differences are small, they are internally consistent and statistically robust after Bonferroni’s conservative correction. These results suggest that the wavelength of 2780 nm cannot directly inhibit CO through photochemistry. CO contains copper centres that absorb infrared light widely between 700 and 1000 nm. Inhibitory photochemical effects on CO have been specifically identified at 750 and 950 nm—well outside the emission band of erbium. Wavelength-dependent CO activity scans identified 750 nm and 950 nm as inhibitory regions, while 810 nm activates the enzyme, modulating the rate of oxygen consumption in a corresponding wavelength-specific manner. At 2780 nm, photon energy is absorbed almost entirely by water, not by CO chromophores, so direct photochemical inhibition of the enzyme is not the real modulation mechanism [22].

Erbium irradiation primarily influences mitochondrial electron flow through thermal effects rather than photochemical reactions [23]. The hydrokinetic ablation mechanism of Er,Cr:YSGG relies on concentrating heat by vaporising water and, at higher energy levels, generating intracellular thermal transients able to disrupt the electron transport chain [23]. The mitochondrial inner membrane’s thermal sensitivity is considered a critical vulnerability [23]. In mitochondrial respiration experiments, CO inhibitors immediately triggered the mitochondrial temperature decrease, illustrating the direct link between CO activity and mitochondrial thermogenesis, highlighting this system’s susceptibility to external thermal disruption [23]. Additionally, at doses below noticeable thermal damage, ROS produced via water photolysis may trigger primary inhibitory effects on signaling pathways [24]. At moderate to high Er,Cr:YSGG doses, singlet oxygen from water photolysis can directly induce mitochondrial permeability transition [24], leading to oscillatory mitochondrial permeability, which may trigger apoptosis initiation [24]. Even transient membrane permeabilization leads to proton gradient dissipation, uncoupling oxidative phosphorylation from electron transport, and collapsing mitochondrial membrane potential (ΔΨm) [24]. These events reduce ATP production and impair RUNX2 transcriptional activity [24]. Laser-induced ROS production can also cause dose-dependent cellular damage, decreasing mitochondrial potential and glutathione levels, demonstrating that ROS can impair mitochondrial membrane potential independently of thermal effects [25,26,27,28,29]. Our results suggest that Er,Cr:YSGG interacts more directly with the mitochondrial electron transport chain via thermal cycling from water absorption, which partially lowers the photoenergetic stimulus needed by near-infrared diode lasers—mainly targeting CO in the red/NIR window—to sustain high NO synthase activity. This reduction was significant only in Erbium 3 and Erbium 6 at higher doses, but not in Erbium 1, indicating a dose-dependent suppression consistent with the hormetic dose–response model introduced earlier. These findings may extend beyond in vitro contexts. NO has a dual role in bone biology: at nanomolar levels, it promotes osteoblast survival, matrix synthesis, and angiogenesis *via* cGMP-dependent pathways [29,30]. However, at the micromolar concentrations produced by iNOS during inflammation, NO inhibits osteoblast differentiation and mediates osteoclast-driven bone resorption [31]. In apical periodontitis, a condition characterised by chronic inflammation and increased iNOS expression [30,32], the ability of higher doses of Er,Cr: YSGG irradiation to moderate diode laser-induced NO elevation offers a clinically relevant means of supporting, rather than disrupting, periapical healing. The ALP results represent the most mechanistically complex findings of this study and may have future clinical significance. First of all, the results revealed a significant increase in ALP activity relative to the control in the diode-exposed osteoblasts (Figure 6). Near-infrared diode laser irradiation has previously been shown to consistently stimulate the expression and activity of ALP in osteoblasts and osteoblast-like cells across multiple wavelengths (808, 830, 940 nm). In line with our results, Medina-Huertas R et al. reported increased ALP levels in MG-63 cells exposed to a 940 nm diode laser, with the greatest increase relative to controls at 1 W/cm^2^ and 3 J [33]. Moreover, Ioanna Bourouni et al., in their study targeting the photomodulatory effects of a diode laser (810 nm) on osteoblasts, HGFs and their co-cultures in vitro, showed that 810-nm laser irradiation positively affected cell proliferation and viability in all experimental groups [34]. Jawad et al. exposed human foetal osteoblasts specifically to 940 nm LLLT diodes at different power settings. All treatment groups showed a significant increase in cell proliferation and differentiation compared to the control group, with both alkaline phosphatase and osteocalcin activity assays confirming increased cell differentiation [35]. Furthermore, the results of Chunyan Ma and collaborators showed that PBM treatment significantly improved the proliferation and osteogenic differentiation of human umbilical cord mesenchymal stem cells [35]. The authors showed that the mechanism underlying the observed effect involved PBM-mediated activation of the upstream Akt signalling pathway, upregulation of P-Akt expression, and increased reactive oxygen species (ROS) levels, thereby inducing mild oxidative stress. Consequently, these molecular events increased ALP activity and the expression of osteogenesis-associated genes, thereby promoting the osteogenic differentiation of human umbilical cord mesenchymal stem cells [35].

**Figure 7:**
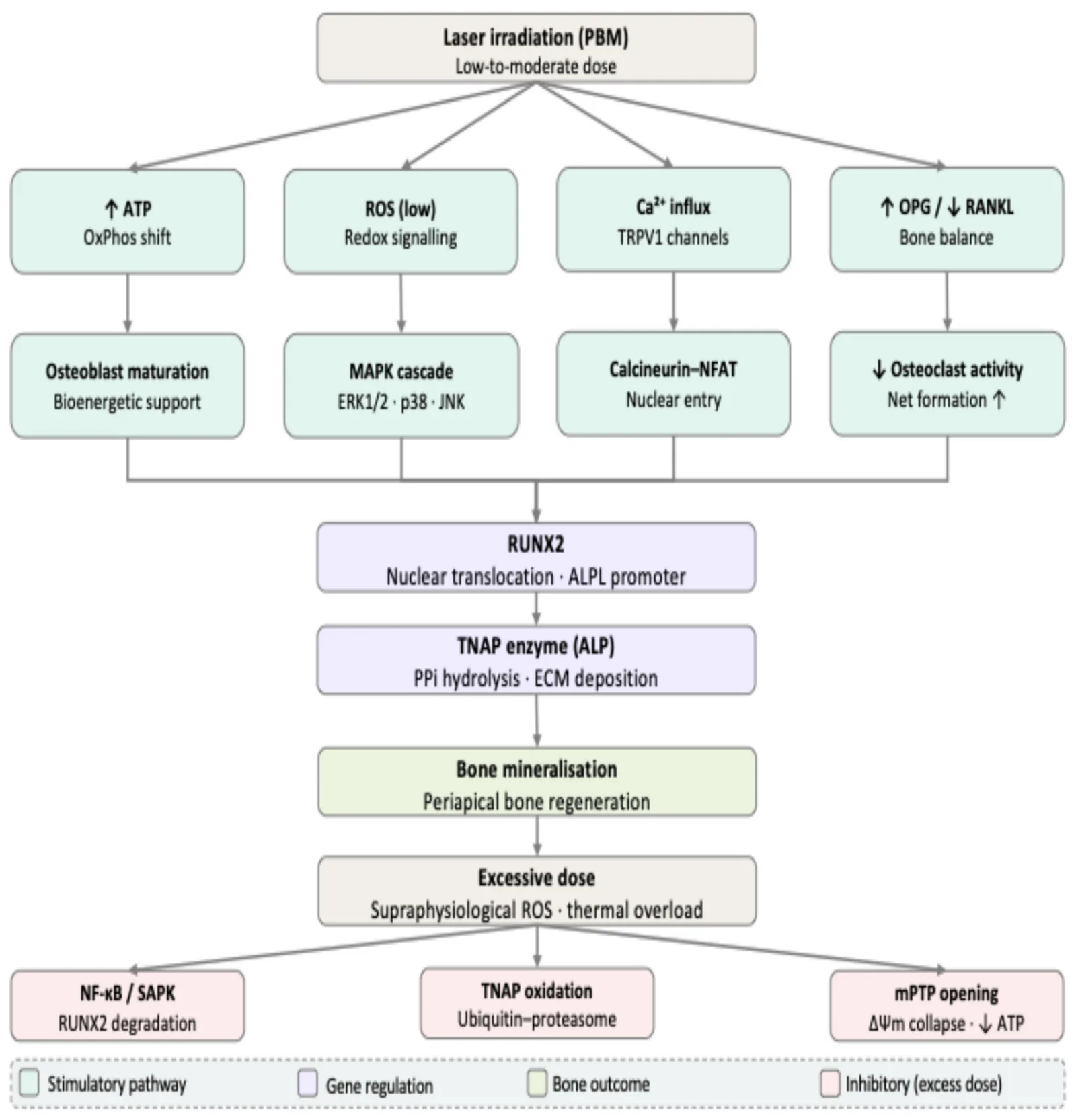
The mechanistic pathways by which photobiomodulation influences alkaline phosphatase expression and activity in osteoblasts.

The molecular means by which photobiomodulation influences the expression and activity of alkaline phosphatase in osteoblasts operate through multiple interconnected pathways, detailed in Figure 7. First, laser-induced increases in ATP availability provide the bioenergetic basis for ATP-intensive consumer processes of matrix synthesis and mineralisation (Figure 7) [35]. Second, controlled ROS generation at low to moderate levels activates MAPK signaling cascades (ERK1/2, p38, JNK), which converge toward RUNX2 phosphorylation and nuclear translocation, directly upregulating the transcription of the ALP gene by binding to osteoblast-specific cis-regulatory elements in the ALPL promoter (the gene encoding ALP) (Figure 7) [9]. Third, laser-mediated calcium influx through light-controlled channels activates calcineurin-NFAT (activated T-cell nuclear factor) signaling, which cooperates with RUNX2 to initiate osteogenic gene expression programs (Figure 7) [9]. Fourth, photobiomodulation modulates the RANKL/OPG ratio (kappa-B ligand/osteoprotegerin nuclear factor receptor activator)—RANKL promotes osteoclastic bone resorption, while OPG acts as a diversion receptor—thus influencing the balance between bone formation and remodelling, which drives the net activity of alkaline phosphatase in the local microenvironment (Figure 7) [9].

However, when light doses exceed the optimal therapeutic level, they activate inhibitory mechanisms due to excessive ROS production (Figure 7). This redox imbalance causes NF-κB to become abnormally elevated, shifting gene expression toward stress and pro-inflammatory directions rather than promoting differentiation. Oxidative modifications of ALP may diminish its enzymatic activity or lead to its breakdown via the ubiquitin–proteasome system. Moreover, overactivation of stress kinases like SAPK/JNK can phosphorylate RUNX2 at inhibitory sites or trigger its degradation, hindering osteoblast differentiation rather than promoting it [9,12,13,14,15]. This complex mechanism may account for the varying effects of different laser parameters (such as wavelength, fluence, irradiance, pulse duration, and repetition rate) on ALP activity, which can range from significant upregulation to complete inhibition, even with similar total energy doses.

The near-complete suppression of ALP activity caused by Er,Cr:YSGG 6 irradiation alone is an interesting finding. For Er,Cr:YSGG 6, our data indicate that the thermal dose may fall within a range that disrupts cellular homeostasis without causing macroscopic ablation. Previous studies on osteoblast ALP activity have identified a key irradiance threshold: no ALP induction at 51 mW/cm^2^, a modest 43% increase at 102 mW/cm^2^, and a nine-fold increase at 204 mW/cm^2^. These results suggest that the ALP response is logarithmic rather than linear with respect to light dose [36,37]. According to the biphasic dose–response model introduced earlier, this aligns with the right side of the hormetic curve: excessive energy exceeds the therapeutic window of photobiomodulation, shifting ROS levels from promoting differentiation to causing oxidative stress. High fluences lead to overproduction of ROS, which can oxidatively modify ALP by damaging its zinc and magnesium binding sites essential for enzyme activity, and can also promote RUNX2 degradation via stress-activated JNK phosphorylation and ubiquitin–proteasome pathways [36,37]. This suppression of the RUNX2 transcriptional pathway decreases ALP gene expression and enzyme protein levels, explaining the very low ALP values observed in our study.

Furthermore, analysing our data (Figure 6), three pairwise contrasts survived Bonferroni correction: Er,Cr:YSGG vs. Diode (*p* = 0.0005, ***), Diode vs. Er,Cr:YSGG 3 + Diode (*p* = 0.032, *), and Diode vs. Er,Cr:YSGG 6 + Diode (*p* = 0.019, *). Both Er,Cr:YSGG 3 + Diode and Er,Cr:YSGG 6 + Diode produced significantly lower ALP values than Diode alone, suggesting that Er,Cr:YSGG irradiation partially counteracts the osteogenic effect of the diode laser. One possible explanation is the competitive interaction between chromophores: applying Er,Cr:YSGG irradiation before or alongside may cause thermomechanical stress, such as temporary intracellular water vaporization, membrane damage, and non-specific protein denaturation. These effects could impair mitochondrial CO and ROS-dependent signaling pathways vital for diode laser photobiomodulation. Alternatively, the thermal effects of sequential dual-wavelength irradiation might trigger heat shock protein (HSP70, HSP90) responses, thereby reorienting cellular resources away from differentiation pathways targeted by photobiomodulation. The dose-dependence pattern observed in our study, with Er,Cr:YSGG 6 + Diode showing slightly lower ALP than Er,Cr:YSGG 3 + Diode, aligns with the thermal dose–response relationship (Figure 6). Moreover, the significant difference between ALP levels of Er,Cr:YSGG 1 and Er,Cr:YSGG 6 dose suggest also a thermal dose–response relationship (Figure 6).

The three significant comparisons, Er,Cr:YSGG vs. Diode (*p* = 0.0005, ***), Diode vs. Er,Cr:YSGG 3 + Diode (*p* = 0.032, *), and Diode vs. Er,Cr:YSGG 6 + Diode (*p* = 0.019, *), taken together might outline the same biological scenario: combining Er,Cr:YSGG irradiation with the diode laser irradiation progressively reduced ALP relative to the diode-alone condition as erbium energy increased (Figure 6).

Taken all together, our findings suggest that the diode laser significantly increases ALP levels compared to controls (statistically significant) (Figure 6) and all Er,Cr:YSGG-alone conditions (although not statistically significant after correction versus control) (Figure 6). This indicates that near-infrared diode irradiation is the more osteogenically favorable wavelength in this experimental setup. This observation is consistent with established photobiomodulation research: wavelengths in the 800–980 nm range (near-infrared window) penetrate tissue with less scattering and are well absorbed by cytochrome c oxidase. This absorption triggers the mitochondrial bioenergetic cascade—increasing mitochondrial membrane potential, ATP production, and transient ROS generation—which promotes MAPK→RUNX2→ALPL upregulation. Conversely, the 2780 nm Er,Cr:YSGG wavelength is mainly absorbed by water and hydroxyapatite rather than mitochondrial chromophores, resulting in thermomechanical rather than photochemical tissue interactions at endodontic energy levels [9,35].

### 3.1. Clinical Implications

Our findings indicate that 810 nm diode laser irradiation alone (6.5 J/cm^2^) yields the highest ALP activity (Figure 6), suggesting that near-infrared photobiomodulation may offer therapeutic advantages, particularly for stimulating osteogenesis during periapical treatments. These results emphasise the importance of conducting further research with more complex models that replicate inflammatory conditions such as apical periodontitis. The consistent maintenance of osteoblast viability and membrane integrity across all protocols (Figure 2, Figure 3 and Figure 4) indicates that Er,Cr:YSGG **(2780 nm)** irradiation at the tested energy levels (1, 3 and 6 J/cm^2^) is likely safe for clinical use. Nevertheless, the notable decrease in ALP levels at Er,Cr:YSGG 6 J/cm^2^ irradiation, despite the absence of evident cytotoxicity, raises concerns that high-energy Er,Cr:YSGG treatments might hinder osteoblast differentiation without causing cell death. This could potentially slow down or reduce bone regeneration after endodontic procedures. Since this functional impairment occurs without cell death, it could be missed if only cell viability is considered, highlighting the need to include differentiation markers in laser biocompatibility assessments. Most importantly, from a practical perspective, the antagonistic interaction observed between combined Er,Cr:YSGG and diode irradiation on ALP activity, compared to diode alone, implies that sequential double-wavelength protocols aimed at promoting periapical healing should consider that prior Er,Cr:YSGG irradiation at higher energy levels (3 and 6 J/cm^2^) might diminish or negate the osteogenic effects of the diode. Carefully optimizing the timing, energy levels, and wavelength ranges of dual-laser protocols is crucial to ensure supporting or enhancing periapical bone regeneration.

### 3.2. Study Limitations

The in vitro monoculture model, while offering a controlled mechanical perspective, does not recapitulate the complexity of the periapical microenvironment, which includes a heterogeneous cellular environment—fibroblasts, immune cells, endothelial cells, and undifferentiated mesenchymal progenitors—embedded in an extracellular matrix scaffold and subjected to inflammatory cytokine gradients. Moreover, the present model did not incorporate bacterial-derived virulence factors (e.g., lipopolysaccharide and other endodontic pathogen-associated molecules), which are key drivers of periapical inflammation and bone resorption; future studies should combine laser irradiation with such stimuli to more closely reproduce the endodontic disease context and evaluate later time points (48–96 h).

The laser spots did not cover the entire well area (≈66% for Er,Cr:YSGG and ≈41% for the diode laser), resulting in non-uniform irradiation of the cell monolayer; since all assays were performed on whole-well samples, the reported effects may underestimate the response of the directly irradiated cells.

The Er,Cr:YSGG exposure was delivered as a single 1 s irradiation; longer or repeated exposures, closer to clinical endodontic protocols, should be evaluated in future studies.

## 4. Materials and Methods

### 4.1. Experimental Design

Human osteoblasts were exposed to eight experimental conditions, with 6 replicates per group for MTT and NTAP (total *n* = 48) and 12 replicates per group for LDH and NO (total *n* = 96). The tested groups were:

• Control—non-irradiated cells;
• Diode—810 nm diode laser irradiation (CW, 0.1 W, 10 s, 1 J, ≈0.66 W/cm^2^, ≈6.5 J/cm^2^)
• Er,Cr:YSGG 1 J, Er,Cr:YSGG 3 J, Er,Cr:YSGG 6 J—Er,Cr:YSGG laser irradiation at three energy levels, 1, 3, and 6 J.
• Er,Cr:YSGG 1 J + Diode, Er,Cr:YSGG 3 J + Diode, Er,Cr:YSGG 6 J + Diode—Er,Cr:YSGG + Diode combinations.

### 4.2. Cell Culture

In the present study, a human fetal osteoblast cell line passage 5 (hOB, Cat. No. 406-05F, Sigma-Aldrich, St. Louis, MO, USA) was employed. The cells were maintained in Osteoblast Basal Medium (OBM, Cat. No. 416-500, Sigma Aldrich, San Diego, CA, USA) according to the manufacturer’s recommendations and incubated at 37 °C in a humidified atmosphere containing 5% CO_2_. For experimental procedures, cells were seeded into 96-well culture plates at a density of 2 × 10^4^ cells per well in a total volume of 300 µL of culture medium and allowed to adhere for 24 h. After cell adhesion, the cells were exposed to laser irradiation under the established experimental conditions. Subsequently, 24 h following laser exposure, cellular responses were comprehensively assessed using the MTT assay, lactate dehydrogenase (LDH) release assay, nitric oxide (NO) quantification, and alkaline phosphatase (ALP) activity evaluation.

### 4.3. Laser Irradiation Protocol

Er,Cr:YSGG laser irradiation was performed using a 2780 nm erbium, chromium-doped yttrium–scandium–gallium–garnet (Er,Cr:YSGG) laser (Waterlase iPlus, Biolase, Irvine, CA, USA) operating in short-pulse “H” mode (60 µs pulse duration) at 15 Hz, with both air and water set to 0, delivered through a 600 µm MZ6 tip (6 mm tip length) mounted on the gold handpiece. Immediately prior to irradiation the culture medium was aspirated, and cells were re-fed immediately afterwards. The handpiece was fixed perpendicular to the plate at a working distance of approximately 1.8 cm from the cell monolayer, producing a laser spot approximately 5.6 mm in diameter (0.25 cm^2^) on the well bottom (verified on paper), centered within each 6.9 mm well. Each well was irradiated for 1 s. Energy densities of 1, 3 and 6 J/cm^2^ were obtained by setting the average power to 0.25, 0.75 and 1.5 W, respectively (pulse energies of 16.7, 50.0 and 100.0 mJ at 15 Hz). Given the manufacturer-stated power accuracy of ±20%, delivered fluences carry a corresponding tolerance.

Photobiomodulation was performed with a multi-wavelength diode laser (Solase Pro, Lazon Medical Laser Co., Ltd., Shenyang, China; 650, 810 and 980 nm; maximum output power at 810 nm: 6 W in continuous-wave mode), of which only the 810 nm wavelength was used. Irradiation was performed in continuous-wave mode at an average power of 0.1 W for 10 s, using the distal end of the fiber-optic delivery cable (handpiece connector), without a handpiece or tip, delivering an energy density of 6.5 J/cm^2^ over an approximately 4.4 mm spot, measured at the level of the cell monolayer, with the culture medium present.

The experimental design comprised eight groups: (1) non-irradiated control; (2) 810 nm diode alone (6.5 J/cm^2^); (3) Er,Cr:YSGG at 1 J/cm^2^; (4) Er,Cr:YSGG at 3 J/cm^2^; (5) Er,Cr:YSGG at 6 J/cm^2^; (6) Er,Cr:YSGG at 1 J/cm^2^ followed immediately by diode irradiation (6.5 J/cm^2^); (7) Er,Cr:YSGG at 3 J/cm^2^ followed immediately by diode irradiation; and (8) Er,Cr:YSGG at 6 J/cm^2^ followed immediately by diode irradiation. In the combined-irradiation groups, cultures were first exposed to the Er,Cr:YSGG laser and subsequently to the diode laser. Non-irradiated osteoblast cultures maintained under identical conditions served as the control group.

### 4.4. Live-Cell Imaging—HoloMonitor M4

Morphological changes, cell proliferation, and migration of hOB cells were observed using the HoloMonitor M4 digital holographic microscope (Phase Holographic Imaging PHI AB, Lund, Sweden). A HoloLid™ (PHI AB) was placed on each plate containing cultured osteoblasts to enhance image quality and reduce evaporation. Time-lapse images were taken every 30 min over 24 h under standard culture conditions (37 °C, 5% CO_2_).

### 4.5. Cell Viability Assay (MTT Assay)

Cell viability of hOB cells was assessed using the MTT assay (Biotium, Cat. No. 30006, Fremont, CA, USA). Twenty-four hours following direct laser irradiation of the hOB cells under the established experimental conditions, 10 µL of MTT reagent was added to each well of the 96-well culture plates containing 100 µL of culture medium (OBM, Cat. No. 416-500, Sigma Aldrich, San Diego, CA, USA) [38,39].

The plates were subsequently incubated at 37 °C for 4 h, allowing metabolically active cells to reduce 3-(4,5-dimethylthiazol-2-yl)-2,5-diphenyltetrazolium bromide (MTT) into insoluble purple formazan crystals. Following the incubation period, the culture medium was carefully removed, the cells were washed with PBS and the resulting formazan crystals were solubilized using isopropanol.

Absorbance was measured using a FLUOstar^®^ Omega multimode microplate reader (BMG LABTECH, Ortenberg, Germany) at a wavelength of 570 nm, while background absorbance was recorded at 630 nm. The measured values were corrected by subtracting the background signal, and cell viability was expressed as a percentage relative to the untreated control group, which was considered to represent 100% viability.

### 4.6. Level of Lactate Dehydrogenase (LDH Assay)

The release of the cytosolic enzyme lactate dehydrogenase (LDH) into the extracellular medium is widely recognized as an indicator of plasma membrane integrity loss and therefore reflects cellular damage and cytotoxicity [17,18]. Cytotoxicity was evaluated by quantifying LDH activity in culture supernatants collected 24 h following laser irradiation of hOB cells using the LDH-Glo™ Cytotoxicity Assay kit (Promega, Cat. No. J2380, Madison, WI, USA). The level of the released LDH was measured. Briefly, 50 µL of culture medium collected from each well was transferred into opaque 96-well plates and combined with 50 µL of LDH-Glo™ Detection Reagent.

Detection Reagent in accordance with the manufacturer’s protocol. Following incubation for 60 min at room temperature, protected from light, luminescence was measured using the MyGLO luminometer (Promega, Madison, WI, USA). The obtained LDH activity values were comparatively analyzed against the untreated control wells to assess the degree of cytotoxicity associated with laser exposure.

### 4.7. Level of Nitric Oxide

Nitric oxide production was indirectly evaluated by quantifying nitrite (NO_2_^−^), a stable oxidation product of nitric oxide, using the Nitric Oxide Assay Kit (Promega, Cat. No. G2930, Madison, WI, USA) in accordance with the manufacturer’s protocol. The analysis was performed on irradiated hOB cells cultured under the established experimental conditions [38,39].

For the assay procedure, 50 µL of culture medium from each sample was dispensed into the wells of a 96-well plate. Subsequently, 50 µL of sulfanilamide reagent was added, and the mixture was incubated for 7 min at room temperature. Thereafter, 50 µL of N-(1-naphthyl) ethylenediamine dihydrochloride (NED) solution was added to the reaction mixture and incubated for an additional 7 min at room temperature, allowing the formation of a chromophoric azo compound.

Optical density was measured using a FLUOstar^®^ Omega microplate reader within the 520–540 nm detection range. Nitric oxide production was determined relative to the control group by comparing the absorbance values obtained for each experimental condition.

### 4.8. Alkaline Phosphatase Assay

Alkaline phosphatase (ALP) activity was quantified using the Alkaline Phosphatase Assay Kit (Sigma-Aldrich, Cat. No. MAK447, St. Louis, MO, USA) in accordance with the manufacturer’s instructions. The measurements were performed on samples derived from the hOB cells, maintained under the experimental conditions described previously.

For the assay procedure, 50 µL cell lysate obtained from irradiated and non-irradiated osteoblast culture wells were dispensed into the wells of a clear flat-bottom 96-well microplate. Subsequently, 150 µL of freshly prepared working reagent, consisting of assay buffer, magnesium acetate, and the p-nitrophenyl phosphate (pNPP) substrate, was added to each well, resulting in a final reaction volume of 200 µL. The plate was gently mixed to ensure homogeneous distribution of the reaction mixture.

Enzymatic activity was kinetically monitored through the detection of p-nitrophenol formation, a yellow chromogenic product generated by the alkaline phosphatase-catalyzed hydrolysis of pNPP. Absorbance values were measured using a FLUOstar^®^ Omega microplate) at 405 nm immediately after reagent addition (T0) and again after 4 min (T4).

ALP activity was calculated based on the difference in absorbance between T4 and T0 according to the formula provided in the manufacturer’s protocol, and the results were expressed as international units per liter (IU/L).

### 4.9. Statistical Analysis

Data analysis was conducted using IBM SPSS Statistics (version 26.0 (IBM, New York, NY, USA)), with a significance level of α = 0.05. Descriptive statistics, including the mean and standard deviation, were calculated for each of the eight experimental groups. The normality of data distribution within each group was tested using the Shapiro–Wilk test, while Levene’s test checked for equal variances. Group comparisons were performed with either parametric or non-parametric methods based on the data assumptions: Welch’s ANOVA followed by Games–Howell post hoc tests for ALP (normal distribution but unequal variances), and the Kruskal–Wallis test with Bonferroni-adjusted pairwise comparisons for LDH, MTT, and NO (due to at least one group violating normality). In all figures, significance is indicated relative to the Diode group, which served as the clinical reference. Significance levels are denoted as * *p* < 0.05, ** *p* < 0.01, and *** *p* < 0.001.

## 5. Conclusions

Within this in vitro study, none of the treatments—Er,Cr:YSGG laser at three energy levels, diode laser, or their combinations—caused statistically significant changes in osteoblast activity, metabolic viability, membrane integrity, lack of production, or ALP activity compared to untreated controls, after Bonferroni correction. Diode-only laser irradiation resulted in the highest osteogenic marker (ALP). Additionally, the combined treatments reduced both the NO-boosting and ALP-enhancing effects of the diode laser in a dose-dependent manner with Er,Cr:YSGG. However, notable differences between treatments indicated that high-energy Er,Cr:YSGG irradiation nearly completely negates the ALP activity of osteoblasts.

These findings suggest overall that Er,Cr:YSGG energy selection in endodontic laser protocols should be calibrated not only for antimicrobial efficacy but also for preservation of periapical osteogenic capacity, and two-wavelength protocols may require careful optimization to avoid antagonizing the osteogenic benefits of near-infrared photobiomodulation.

## Data Availability

The original contributions presented in this study are included in the article. Further inquiries can be directed to the corresponding authors.

## References

[1] Sánchez-Torres A., Sánchez-Garcés M.Á., Gay-Escoda C. (2014). Materials and prognostic factors of bone regeneration in periapical surgery: A systematic review. Med. Oral Patol. Oral Cir. Bucal.

[2] Luo X., Wan Q., Cheng L., Xu R. (2022). Mechanisms of bone remodeling and therapeutic strategies in chronic apical periodontitis. Front. Cell. Infect. Microbiol..

[3] Soldatos N., Romanos G.E., Michaiel M., Sajadi A., Angelov N., Weltman R. (2018). Management of Retrograde Peri-Implantitis Using an Air-Abrasive Device, Er,Cr:YSGG Laser, and Guided Bone Regeneration. Case Rep. Dent..

[4] Butucescu M., Voicu Balasea B., Imre M., Pana I., Radulescu R., Duica F., Dinu M., Rus F., Parau A.C., Kiss A.E. (2026). Biological response of human osteoblastscultured on magnesium-doped hydroxyapatite coatings on Ti6Al7Nb alloy substrates. Ceram. Int..

[5] Sekaran S., Vimalraj S., Thangavelu L. (2021). The Physiological and Pathological Role of Tissue Nonspecific Alkaline Phosphatase beyond Mineralization. Biomolecules.

[6] Oliveira F.A., Matos A.A., Matsuda S.S., Buzalaf M.A., Bagnato V.S., Machado M.A., Damante C.A., Oliveira R.C., Peres-Buzalaf C. (2017). Low level laser therapy modulates viability, alkaline phosphatase and matrix metalloproteinase-2 activities of osteoblasts. J. Photochem. Photobiol. B.

[7] Fahim S.Z., Ghali R.M., Hashem A.A., Farid M.M. (2024). The efficacy of 2780 nm Er,Cr;YSGG and 940 nm Diode Laser in root canal disinfection: A randomized clinical trial. Clin. Oral Investig..

[8] Al-Karadaghi T.S., Franzen R., Jawad H.A., Gutknecht N. (2015). Investigations of radicular dentin permeability and ultrastructural changes after irradiation with Er,Cr:YSGG laser and dual wavelength (2780 and 940 nm) laser. Lasers Med. Sci..

[9] Hamblin M.R. (2018). Mechanisms and Mitochondrial Redox Signaling in Photobiomodulation. Photochem. Photobiol..

[10] Moisa M., Voicu Balasea B., Imre M., Radulescu R., Duica F., Rus F., Pana I., Muscurel C., Popa A., Ripszky A. (2025). Insights into the biocompatibilityof human gingival fibroblasts cultured on a hydroxyapatite-coated new Ti-Nb Alloy. New perspectives for dentistry?. Ceram. Int..

[11] Murata Y., Osakabe M. (2013). The Bunsen-Roscoe reciprocity law in ultraviolet-B-induced mortality of the two-spotted spider mite Tetranychus urticae. J. Insect Physiol..

[12] Sies H., Jones D.P. (2020). Reactive oxygen species (ROS) as pleiotropic physiological signalling agents. Nat. Rev. Mol. Cell Biol..

[13] Sies H., Belousov V.V., Chandel N.S., Davies M.J., Jones D.P., Mann G.E., Murphy M.P., Yamamoto M., Winterbourn C. (2022). Defining roles of specific reactive oxygen species (ROS) in cell biology and physiology. Nat. Rev. Mol. Cell Biol..

[14] Enasescu D.S., Voicu-Balasea B., Rus-Hrincu R.-H., Radulescu R., Popa A., Moisa M.R., Coculescu B.-I., Imre M.M., Pituru S.M. (2024). Exploring comparatively the cytotoxicity of milled and 3D-printed PMMA-based resins to oral squamous cancer cells-what signals could the akt/mtor pathway send us through its downstream effector GSK-3?. Rom. J. Oral Rehabil..

[15] Alhaj Sulaiman A., Katanaev V.L. (2025). Beyond Antioxidants: How Redox Pathways Shape Cellular Signaling and Disease Outcomes. Antioxidants.

[16] Hamblin M.R. (2017). Mechanisms and applications of the anti-inflammatory effects of photobiomodulation. AIMS Biophys..

[17] Ponnusamy S., Amin M., Bhat A., Garczynski S., Rahman S.U., Rasam S., Govindool S.R., Khan I., Arany P. (2026). Photobiomodulation Activates Coordinated Signaling Networks to Modulate Inflammation, Adaptive Stress, and Tissue Healing via Redox-Mediated NFkappaB-TGF-beta1-ATF-4 Axis. Cells.

[18] Arany P.R. (2022). Photobiomodulation-Activated Latent Transforming Growth Factor-beta1: A Critical Clinical Therapeutic Pathway and an Endogenous Optogenetic Tool for Discovery. Photobiomodul. Photomed. Laser Surg..

[19] Sippus J.H., Capitanio M., Mutluay M.M., Franzen R., Tezvergil-Mutluay A. (2025). Evaluation of Er:YAG, Er,Cr:YSGG and diode lasers irradiation on radicular dentine fatigue strength using modified endodontic laser tips. Biomater. Investig. Dent..

[20] Franceschini K.A., Silva-Sousa Y.T., Lopes F.C., Pereira R.D., Palma-Dibb R.G., de Sousa-Neto M.D. (2016). Bond strength of epoxy resin-based root canal sealer to human root dentin irradiated with Er,Cr:YSGG laser. Lasers Surg. Med..

[21] Papoulidou I., Dionysopoulos D., Mourouzis P., Naka O., Sarris K., Baldi A., Scotti N., Tolidis K. (2023). Evaluation of Er,Cr:YSGG laser technique for fiber post removal of endodontically treated teeth using micro-computed tomography. Microsc. Res. Tech..

[22] Sanderson T.H., Wider J.M., Lee I., Reynolds C.A., Liu J., Lepore B., Tousignant R., Bukowski M.J., Johnston H., Fite A. (2018). Inhibitory modulation of cytochrome *c* oxidase activity with specific near-infrared light wavelengths attenuates brain ischemia/reperfusion injury. Sci. Rep..

[23] Macherel D., Haraux F., Guillou H., Bourgeois O. (2021). The conundrum of hot mitochondria. Biochim. Biophys. Acta Bioenerg..

[24] Novikova I.N., Potapova E.V., Dremin V.V., Dunaev A.V., Abramov A.Y. (2022). Laser-induced singlet oxygen selectively triggers oscillatory mitochondrial permeability transition and apoptosis in melanoma cell lines. Life Sci..

[25] Saenko Y.V., Glushchenko E.S., Zolotovskii I.O., Sholokhov E., Kurkov A. (2016). Mitochondrial dependent oxidative stress in cell culture induced by laser radiation at 1265 nm. Lasers Med. Sci..

[26] Amaroli A., Pasquale C., Zekiy A., Utyuzh A., Benedicenti S., Signore A., Ravera S. (2021). Photobiomodulation and Oxidative Stress: 980 nm Diode Laser Light Regulates Mitochondrial Activity and Reactive Oxygen Species Production. Oxid. Med. Cell. Longev..

[27] Zhang L., Wang X., Cueto R., Effi C., Zhang Y., Tan H., Qin X., Ji Y., Yang X., Wang H. (2019). Biochemical basis and metabolic interplay of redox regulation. Redox Biol..

[28] Sokolovski S.G., Rafailov E.U., Abramov A.Y., Angelova P.R. (2021). Singlet oxygen stimulates mitochondrial bioenergetics in brain cells. Free Radic. Biol. Med..

[29] Navarro G., Gómez-Autet M., Morales P., Rebassa J.B., Llinas Del Torrent C., Jagerovic N., Pardo L., Franco R. (2024). Homodimerization of CB_2_ cannabinoid receptor triggered by a bivalent ligand enhances cellular signaling. Pharmacol. Res..

[30] Nascimento M.H.M., Pelegrino M.T., Pieretti J.C., Seabra A.B. (2020). How can nitric oxide help osteogenesis?. AIMS Mol. Sci..

[31] Butucescu M., Imre M., Rus-Hrincu F., Voicu-Balasea B., Popa A., Moisa M., Ripszky A., Neculau C., Pituru S.M., Pârvu S. (2024). Cell-Type-Specific ROS–AKT/mTOR–Autophagy Interplay—Should It Be Addressed in Periimplantitis?. Diagnostics.

[32] Silva M.J., Sousa L.M., Lara V.P., Cardoso F.P., Júnior G.M., Totola A.H., Caliari M.V., Romero O.B., Silva G.A., Ribeiro-Sobrinho A.P. (2011). The role of iNOS and PHOX in periapical bone resorption. J. Dent. Res..

[33] Medina-Huertas R., Manzano-Moreno F.J., De Luna-Bertos E., Ramos-Torrecillas J., García-Martínez O., Ruiz C. (2014). The effects of low-level diode laser irradiation on differentiation, antigenic profile, and phagocytic capacity of osteoblast-like cells (MG-63). Lasers Med. Sci..

[34] Bourouni I., Kyriakidou K., Fourmousis I., Vrotsos I.A., Karoussis I.K. (2021). Low level laser therapy with an 810-nm diode laser affects the proliferation and differentiation of premature osteoblasts and human gingival fibroblasts in vitro. J. Lasers Med. Sci..

[35] Manzano-Moreno F.J., Medina-Huertas R., Ramos-Torrecillas J., García-Martínez O., Ruiz C. (2015). The effect of low-level diode laser therapy on early differentiation of osteoblast via BMP-2/TGF-β1 and its receptors. J. Craniomaxillofac. Surg..

[36] Ma C., Ye Y., Shi X., Li N., Chen Y., Shi X., Chen H. (2025). Photobiomodulation promotes osteogenic differentiation of mesenchymal stem cells and increases P-Akt levels in vitro. Sci. Rep..

[37] Lian S., Wang T., Li J., Yang Q., Lu C. (2024). Tauroursodeoxycholic Acid Mitigates Oxidative Stress and Promotes Differentiation in High Salt-Stimulated Osteoblasts via NOX1 Mediated by PGC-1alpha. Discov. Med..

[38] Popa A., Imre M., Pițuru S.M., Voicu-Bălașea B., Cernega A., Rădulescu R.V., Rus F., Trușcă R., Musteanu M., Andronescu E. (2026). Physicochemical Characterization and In Vitro Biocompatibility of Epoxy- and Silicone-Based Endodontic Materials. Materials.

[39] Popa A., Radulescu R.V., Rus F., Vasilescu V.G., Ciocan L.T., Musteanu M., Imre M., Pituru S., Cernega A., Ripszky A. (2026). Dental Glass Ionomer Cement for Root Perforation Management: Physicochemical Characteristics and In Vitro Cell Response. Dent. J..

